# Effects of Scanning Speed on the Microstructure and Wear Properties of Rockit 606 Coating Layer by Disk Laser Cladding

**DOI:** 10.3390/ma17194758

**Published:** 2024-09-27

**Authors:** Tianqing Li, Zhiwei Bi, Yucheng Lei

**Affiliations:** School of Materials Science and Engineering, Jiangsu University, Zhenjiang 212013, China

**Keywords:** laser cladding, Rockit 606, scanning speed, microstructure, wear resistance

## Abstract

Improving the wear resistance and corrosion resistance of 60Si2Mn steel is an important issue in agricultural machinery. A Rockit 606 coating layer may exhibit excellent performance in wear resistance and corrosion resistance. However, there are very a few public reports and articles involving the topic of a Rockit 606 laser cladding layer on a steel 60Si2Mn surface. It is of great importance to research Rockit 606 laser cladding layers. This work focuses on studying the microstructure and properties of Rockit 606 coating layers with different scanning speeds by disk laser cladding. Firstly, the laser cladding platform was designed and set up. Secondly, the laser cladding parameters were designed, and then the laser cladding experiment was conducted, and the Rockit 606 coating layers were obtained. And finally, the microstructure, phase distribution, corrosion resistance, surface hardness, and wear resistance of the coating layers were measured and analyzed. A scanning electron microscope (SEM), X-ray diffractometer (XRD), electrochemical workstation, and microhardness tester were used in this work. It was found that the microstructure Rockit 606 alloy coating consists of γ-Fe, V_8_C_7_, and Cr_7_C_3_. The microhardness of the Rockit 606 alloy coatings decreased with an increase in the scanning speed. When the scanning speed was 4 mm/s, the highest microhardness value reached 867.2 HV, which is about three times of that of the substrate. The average coefficients of friction (COFs) of the coatings decreased with an increase in the scanning speed, which led to the corresponding decrease of the wear rate. When the scanning speed was 4 mm/s, the wear behavior of the coating was mainly oxidative wear and a small amount of adhesive wear. The self-corrosion current density of the coatings prepared by laser cladding in a 3.5 wt.% NaCl solution is one order of magnitude lower than that of the substrate, indicating that the coatings have better corrosion resistance properties.

## 1. Introduction

In the field of railway vehicles [1] and agricultural machinery [2], steel 60Si2Mn is one of the alternative materials. Steel 60Si2Mn is a kind of low alloy steel with high strength, good hardenability, a large load-bearing capacity, and low cost. The failure of mechanical components made by steel 60Si2Mn mainly stems from wear and corrosion [3,4], which limits the use of 60Si2Mn. Replacing 60Si2Mn with materials with higher wear resistance and corrosion resistance will incur significant costs. Therefore, surface coating and modification of steel 60Si2Mn is an effective solution to improve wear resistance and corrosion resistance [5]. There are several surface coating and modification methods that can improve the surface properties of agricultural machinery, including thermal spraying [6], plasma spraying [7], chemical vapor deposition (CVD) [8], selective laser melting (SLM) [9], physical vapor deposition (PVD) [10], high velocity oxygen fuel (HVOF) spraying [11], and laser cladding technology. Laser cladding technology (LC) is an advanced surface modification technique that utilizes high-energy laser beams to melt cladding materials and solidify them on the surface of the substrate, and it forms a cladding coating with high service performance [12]. Compared to other surface strengthening technologies, laser cladding technology offers several advantages [13,14]. It can enhance the comprehensive properties of materials’ surfaces, such as wear and corrosion resistance, without altering or affecting the original properties of the substrate [14,15]. Using laser cladding technology to deposit high-melting point, high-hardness, and corrosion-resistant high-entropy alloys on the steel surface is an innovative method to improve the hardness, wear resistance, and corrosion resistance of the steel surface [16]. Ma et al. [17] prepared high-entropy FeCoCrNiAl alloy coatings with different tungsten contents using laser cladding technology. The results showed that the average hardness of FeCoCrNiAlW0.8 reached 756.83 HV0.2, which is 2.97 times that of the 45 steel matrix. Meanwhile, the friction coefficient of the coating gradually decreases, indicating an increase in wear resistance. The corrosion resistance of the coating is significantly improved after adding W. Kim et al. [18] studied the effects of the laser power, melting rate, and powder feeding rate on the geometric shape, microstructure, and hardness of the melting layer. The results indicate that the microstructure and hardness of the laser cladding layer depend on process parameters and powder characteristics, while the wear resistance of the coating must consider the wear mechanism, including the hardness and characteristics of the powder used. Adamiak et al. [19] deposited a nickel-based powder composed of NiSiB + 60% WC onto structural steel substrates using laser cladding and plasma powder transfer arc welding. The findings of this study indicate that both methods are promising for anti-wear applications due to their wear-resistant properties and metallurgical bonding to the substrate. The laser cladding method may be advantageous in applications that require lower dilution and a larger HAZ. Liu et al. [20] prepared Inconel 625 alloy laser cladding layers with different Fe contents on the surface of 20 steel and obtained a reasonable dilution rate in the laser cladding layer. The results indicate that the grain size of the cladding layer becomes coarser with an increase in the Fe content, and the grain orientation difference increases first and then decreases with an increase in the Fe content. The hardness, high-temperature wear resistance, and high-temperature corrosion resistance gradually decreased. Basiru et al. [21,22] observed that adding a Cr_3_C_2_ phase and VC phase to iron-based coatings improved the wear and corrosion resistance of tools significantly under harsh abrasive and impact conditions. Rockit 606 powder is a kind of iron-based powder containing Cr. If Rockit 606 powder is used as a coating material in laser cladding, the Rockit 606 coating layer may exhibit excellent performance in wear resistance and corrosion resistance. However, there are very a few public reports and articles involving the topic of Rockit 606 laser cladding layer on a steel 60Si2Mn surface.

Therefore, this work focuses on studying the microstructure and properties of a Rockit 606 coating layer with different scanning speeds by laser cladding, which has not been extensively studied in previous works. What is the wear resistance performance of the Rockit 606 coating layer? And what effects does the scanning speed produce on phase distribution and corrosion resistance? In order to know the answers to the above questions, the following work was carried out. Firstly, the laser cladding platform was designed and set up. Secondly, the laser cladding parameters were designed, and then the laser cladding experiment was conducted, and the Rockit 606 coating layers were obtained. And finally, the microstructure, phase distribution, corrosion resistance, surface hardness, and wear resistance of the coating layers were measured and analyzed. How the scanning speed affects the phase distribution and mechanical properties of the coating was studied. This study will enrich the theoretical foundation of laser cladding with Rockit 606 and offer practical guidance for enhancing the coating process of Rockit 606 on 60Si2Mn steel, potentially leading to better industrial applications of this material.

## 2. Materials and Methods

### 2.1. Materials

Hot-rolled 60Si2Mn was selected as the experimental substrate, with size of 100 × 50 × 12 mm^3^. The surface of the substrate was ground with different grades of sandpaper (from 80# to 800#) to remove impurities, and then cleaned with anhydrous ethanol and acetone. The Rockit 606 Fe-based alloy powder produced by Höganäs Company (Helsingborg, Sweden) was chosen as the coating material. The chemical composition of 60Si2Mn and Rockit 606 is shown in Table 1. Before laser cladding, the alloy powder was dried in an oven at a temperature of 100 °C for 3 h.

### 2.2. Laser Cladding

**Figure 1:**
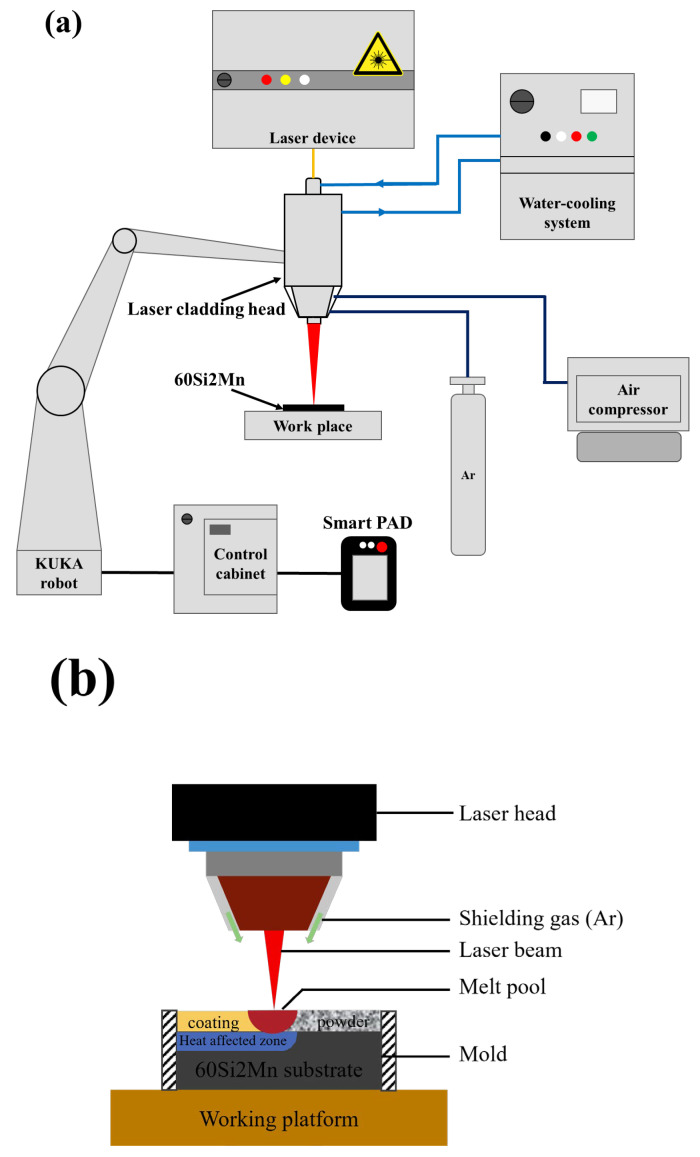
(**a**) Experimental setup diagram of laser cladding system; (**b**) schematic diagram of laser cladding.

The equipment for this study was an integrated system (shown schematically in Figure 1), which mainly consists of laser device (TruDisk 6002, Trumpf Company, Ditzingen, Germany), robot (H60A, KUKA AG, Augsburg, Germany), laser cladding head, air compressor, shielding gas system, and water-cooling system. The maximum output power of the laser device is 6000 W, and the wavelength of disk laser was 1030 nm. The fiber with core diameter of 200 μm was used to transferring laser energy from Trumpf Disk 6002 laser device to laser cladding head. The defocusing amount was 0 mm to ensure the coincidence of the deposition plane and the laser focal plane and to improve the laser energy and powder capture efficiency. The inert gas Ar was used as the shielding gas, with a purity of 99.99%. The shielding gas flow rates was 10 L/min. The preplacement was conducted by evenly spreading the Rockit 606 powder on the surface of the substrate steel plate, controlling the preset layer thickness at 1.5 mm using a mold. Excess powder was removed with a glass rod. The powder was then compacted by 90–100 kg load for about 5 min. And then the powder is melted by the laser beam, and the cladding coating is formed after solidification. The experimental parameters used in the study are presented in Table 2.

### 2.3. Sample Characterization

The cross-sectional metallographic samples of the coating were prepared by precision wire cutting, following ASTM E3-11 [23] guidelines for the preparation of metallographic specimens, to avoid any damage or deformation. These samples were then subjected to an etching process to reveal the microstructure. The etching solution was a metallographic etchant consisting of FeCl_3_ (1 g), HCl (3 mL), and H_2_O (20 mL). Etching was carried out at room temperature by immersing the cross-sections for a controlled duration of 30–60 s, depending on the reaction speed with the material, followed by immediate rinsing with distilled water and alcohol to stop the etching process. After etching, the samples were dried to prevent oxidation or further corrosion. The microstructure of the etched cross-sections was examined using an emission scanning electron microscope (SEM, NovaNamo450, FEI Company, Hillsboro, OR, USA), adhering to ASTM E766 [24] for imaging metallographic specimens.

The phase composition of the cladding coatings was identified using an X-ray diffractometer (XRD, SmartLab, Rigaku Corporation, Tokyo, Japan). The measurements were conducted over a scanning range of 10° to 100° 2θ with a scanning speed of 5°/min, in accordance with ASTM E3294 [25], the standard for qualitative phase analysis using XRD.

The microhardness of the coating was measured using a Vickers hardness tester (FM-ARS900, Future-Tech, Tokyo, Japan), following ASTM E384 [26] for micro-indentation hardness testing. The measurement points were spaced at 100 μm intervals, with a load of 300 gf and a dwell time of 15 s. The hardness value at the same distance is the average of three indents.

The wear resistance of the coatings at room temperature was evaluated using an high-temperature friction (HT-1000, Zhongke Kaihua Technolog Company, Lanzhou, China) wear tester, based on ASTM G99 [27] for pin-on-disk wear testing. The test configuration consisted of a Si_3_N_4_ ball, 4 mm in diameter, sliding against the cladding surface under an applied load of 10 N. The sliding speed was set at 200 r/min, and the total sliding time was 30 min, corresponding to a sliding distance of approximately 75 m. During the test, the friction force and real-time changes in the friction coefficient were continuously recorded by the software, which automatically generated a friction coefficient curve for the entire duration of the test. The average friction coefficient was calculated as the mean value over the total sliding time. The entire wear trajectory was observed using laser confocal microscopy (OLS4100, Olympus Corporation, Tokyo, Japan) and used to calculate the wear volume. Additionally, SEM was employed to observe the wear morphology, providing insight into the wear mechanisms.

Electrochemical testing was conducted at room temperature using an electrochemical workstation (1260A + 1287A, Solartron Metrology, Izmir, Türkiye) in a conventional three-electrode cell setup, following ASTM G5 [28] for potentiodynamic polarization measurements. The reference electrode was a saturated calomel electrode (SCE), the counter electrode was a platinum foil, and the working electrodes were prepared from 60Si2Mn or Rockit 606 alloy coatings. The working electrode surface was carefully polished, following ASTM E407 [29] for surface preparation, and then sealed with epoxy resin, leaving an exposed working area of 1.0 cm². The electrolyte used for the tests was a 3.5 wt.% NaCl solution to simulate a corrosive environment, in accordance with ASTM G31 [30] for laboratory immersion corrosion testing. The polarization measurements were conducted at a scan rate of 1 mV/s, with a potential scan range from −2 V to +1 V.

## 3. Results and Discussion

### 3.1. Macroscopic Morphology Analysis

**Figure 2:**
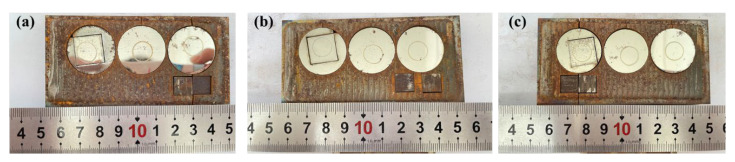
Surface macroscopic morphology of Rockit 606 alloy coatings at different scanning speeds (**a**) 4, (**b**) 6, (**c**) 8 mm/s.

**Figure 3:**
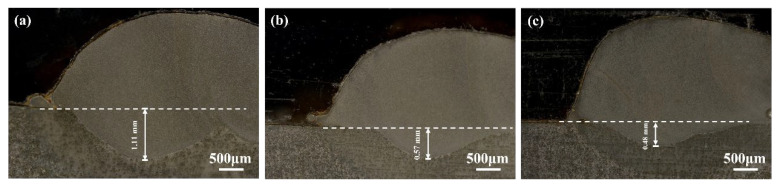
Penetration of Rockit 606 alloy coatings at different scanning speeds (**a**) 4, (**b**) 6, (**c**) 8 mm/s.

Figure 2 shows the macroscopic surface morphology of the Rockit 606 coating. As shown in the figure, the surface topography of the Rockit 606 coating generally demonstrates good formation quality. Figure 3 presents the cross-section between the substrate and the coating. For the samples prepared at scanning speeds of 4, 6, and 8 mm/s, the penetration depths were 1.11, 0.57, and 0.48mm, respectively. As the scanning speed increases, the penetration depth decreases.

### 3.2. Microstructure Evaluation

**Figure 4:**
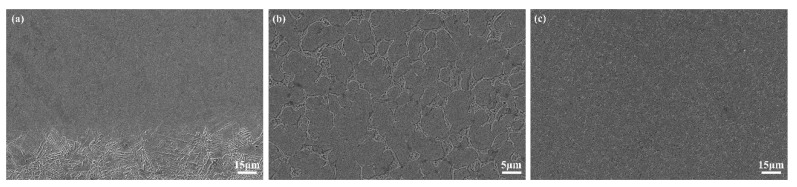
SEM images of the cladding cross-section; (**a**) combining area, (**b**) central region, and (**c**) top region.

Figure 4 shows the microstructure of the laser cladding coatings at different positions. It is found that the microstructure of the Rockit 606 alloy coating at different positions exhibits different features. The microstructure presented by SEM at the combining area between the coating and the substrate shows the characteristics of rapid solidification, as shown in Figure 4a. The microstructure at the central section of the cladding coating consists of dendritic grains (including some cellular grains), as shown in Figure 4b. The microstructure at the top of the cladding coating consists of the equiaxed grains, as shown in Figure 4c.

**Figure 5:**
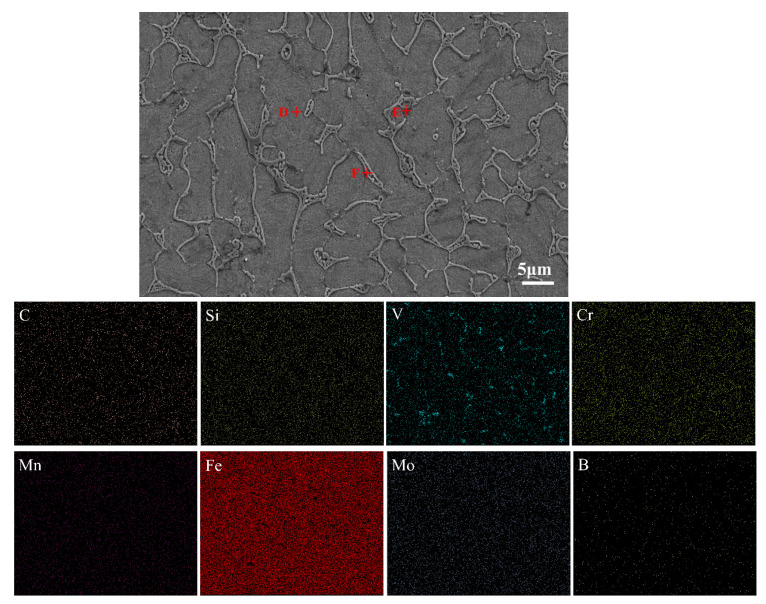
SEM image and EDS mapping scan of Rockit 606 alloy coating.

The composition of the Rockit 606 alloy coating was measured using an energy dispersive spectrometer (EDS) attached to a scanning electron microscope. Figure 5 shows the element distribution shown via an EDS mapping scan. A non-uniform distribution of V is found in the Rockit 606 alloy coating. To quantitatively describe the distribution characteristics of elements in the coating, the three points D, E, and F were selected for spot scanning tests using an EDS. Table 3 is the measured chemical compositions of Points D~F. From Figure 5, it can be inferred that the coating mainly consists of dendrites, interdendritic eutectics, and dispersed intermetallic compounds. Point D is in the interdendritic region rich in Fe. Point E is in the dendrite arm. The EDS results of Point E showed that the primary elements in the dendrite region were Cr, V, and C. Point F is at the intermetallic compounds’ parts. This region primarily contained V and C. It is interesting to reveal what the intermetallic compounds are.

**Figure 6:**
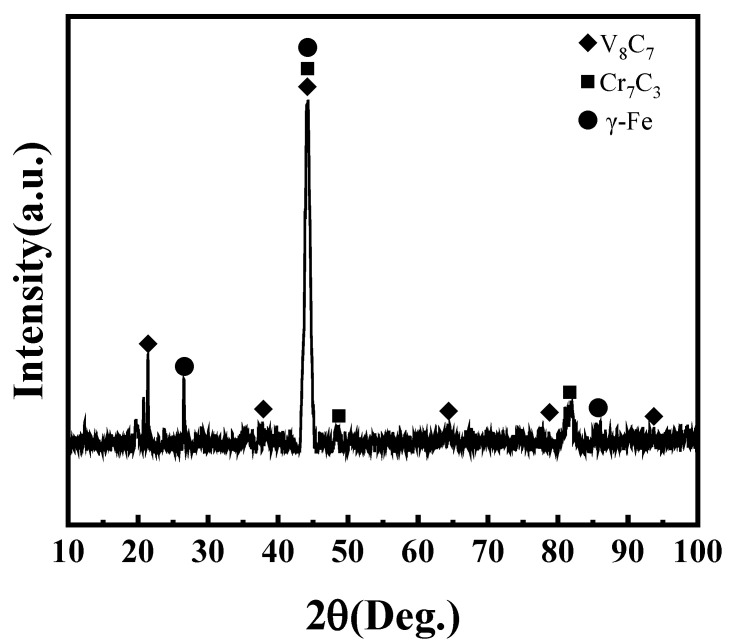
XRD analysis of coating.

In order to know what the intermetallic compounds are, an X-ray diffraction (XRD) analysis was conducted. Figure 6 shows the XRD results and analysis of the Rockit 606 alloy coating. The results show that the primary phases in the coating were γ-Fe, V_8_C_7_, and Cr_7_C_3_. Combining the EDS and XRD analyses, it can be concluded that the dendrites are composed of Cr_7_C_3_ and V_8_C_7_, and the interdendritic region is a γ-Fe solid solution, and dispersed intermetallic compounds are composed of V_8_C_7_.

**Figure 7:**
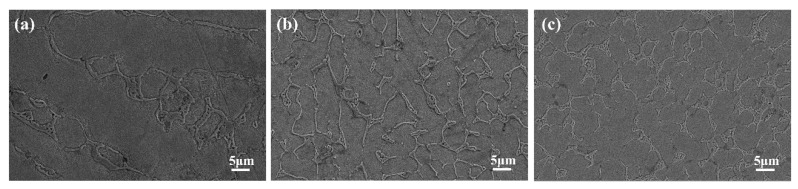
SEM images of coating at different scanning speeds; (**a**) 4, (**b**) 6, (**c**) 8 mm/s.

The effects of scanning speed on the microstructure of the Rockit 606 alloy coating were studied. Figure 7 shows the microstructure of coatings with different scanning speeds as shown by SEM. Black dot-like V_8_C_7_ and a network-like structure of Cr_7_C_3_ and V_8_C_7_ attached to dendrites can be observed. Changes in scanning speed affect the heat input and thermal gradient, which in turn influence the microstructure [31]. The eutectic V_8_C_7_ precipitates at around 1650 °C, and Cr-rich carbide begins to form below the vanadium carbide separation line [32]. It is worth emphasizing that at a low scanning speed of 4 mm/s, the higher heat input per unit area forms a larger melt pool due to the extended interaction time between the laser and the material. Moreover, the higher heat input facilitates the melting of more V and C, resulting in the formation of more dispersed V_8_C_7_ and Cr_7_C_3_ in the coating. This will increase hardness inevitably and may enhance other mechanical properties such as wear resistance. Discontinuous M_7_C_3_ (e.g., Cr_7_C_3_) is tougher than continuous M_3_C [33]. Additionally, the low scanning speed increases the temperature–time state, allowing the sample to reach the required formation temperature of the strengthening phases. In contrast, as the scanning speed increases, the heat input decreases. This reduces the amount of melting and the formation of carbides, resulting in lower hardness and wear resistance [34]. The higher scanning speeds reduce the time for solidification and phase formation, leading to less formation of hard phases of V_8_C_7_.

### 3.3. Microhardness

**Figure 8:**
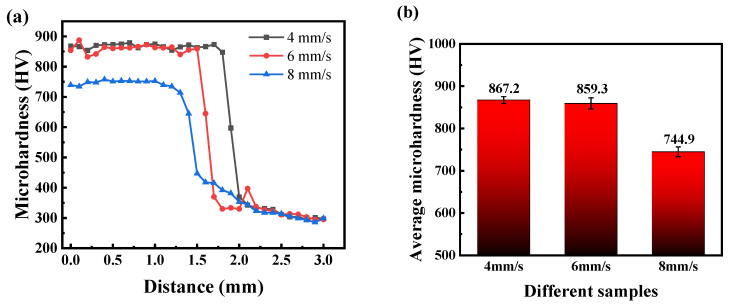
Microhardness of the coating; (**a**) the hardness distribution from the surface to the substrate direction in the coating, and (**b**) the average hardness of the coating area.

The microhardness test results from the substrate to the coating are shown in Figure 8. It can be observed that the microhardness value exhibits an increasing trend from the substrate to the heat-affected zone and then to the coating. The hardness values of the coating are all higher than those of the substrate, while the heat-affected zone’s hardness values fall between the value of the substrate hardness and the coating hardness. Two factors may result in the increasing hardness. One factor is the solid solution strengthening provided by the γ-Fe solid solution in the coating, and the other is the dispersion strengthening effect of the V_8_C_7_ and Cr_7_C_3_ phases. Additionally, laser cladding is a rapid melting and solidification welding process, resulting in fine grains in the coating that lead to grain refinement and, consequently, further increase the coating’s hardness. The main phase in the heat-affected zone is γ-Fe, with a lower content of hard phases, resulting in a decrease in hardness compared to the coating. The average hardness of the coating gradually declines with an increase in scanning speed. When the scanning speed is 4 mm/s, the coating achieves the highest microhardness of 867.2 HV, which is about three times that of the 60Si2Mn substrate.

### 3.4. Wear Performance

**Figure 9:**
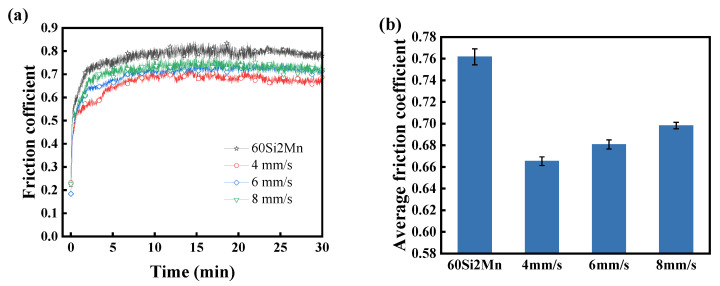
Friction coefficient analysis of Rockit 606 alloy coating and 60Si2Mn: (**a**) friction coefficient change curve, (**b**) average coefficient of friction.

Figure 9a presents the friction coefficient curves of the Rockit 606 alloy coating and the substrate. In the friction process of the substrate, the friction coefficient is less steady than the coating coefficients. The coating undergoes a good running-in stage before entering a stable wear stage, resulting in a lower wear rate. To observe the effect of different scanning speeds on the coating’s friction coefficient, the average values of friction coefficients from the substrate and the coating were calculated and plotted, as shown in Figure 9b. The average friction coefficient of the substrate is 0.76, while the average friction coefficients of the coatings prepared at different scanning speeds are 0.67, 0.68, and 0.69. The average friction coefficients of coatings prepared at different scanning speeds are all lower than that of the substrate, and the average friction coefficient increases with an increase in scanning speed. This improvement is due to the higher presence of hard phase particles (V_8_C_7_, Cr_7_C_3_) in the coating at lower scanning speeds. The hard phase particles (V_8_C_7_, Cr_7_C_3_) effectively separate the friction surfaces and reduce shear and plowing forces between the friction surfaces, and thereby effectively reduce the friction coefficient and coating wear.

**Figure 10:**
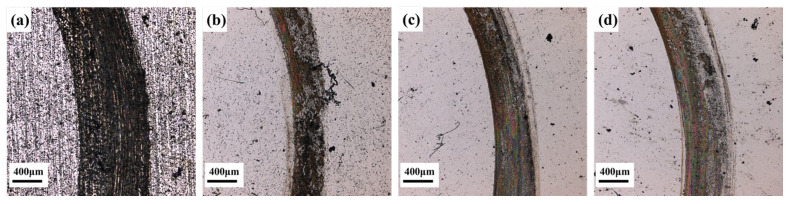
Macroscopic morphology of coatings and 60Si2Mn friction wear surfaces; (**a**) the substrate (**b**) 4, (**c**) 6, (**d**) 8 mm/s.

**Figure 11:**
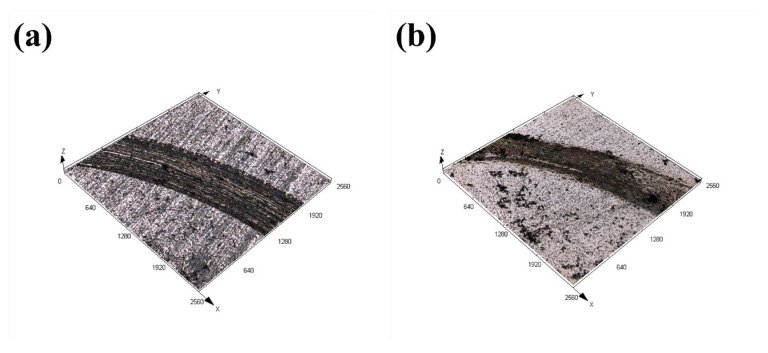

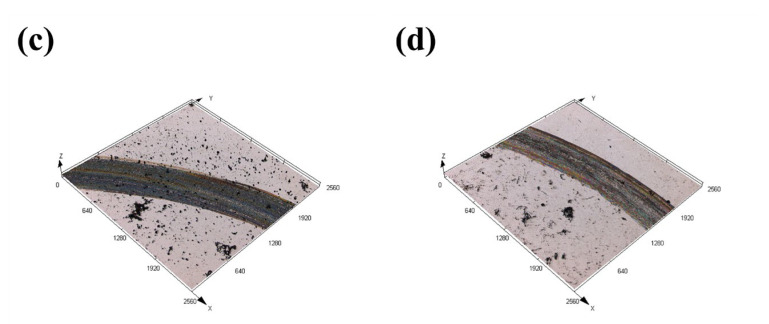
Localized three-bit morphology after frictional wear of coating and 60Si2Mn; (**a**) the substrate (**b**) 4 mm/s, (**c**) 6 mm/s, (**d**) 8 mm/s.

**Figure 12:**
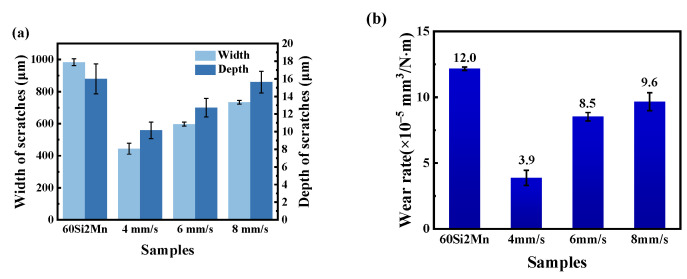
Wear characteristics of laser-fused coatings and substrate: (**a**) width and depth of wear marks; (**b**) wear rate.

Figure 10 and Figure 11 present the macroscopic features and 3D profiles of the wear tracks on the coating and substrate. The characteristics of the wear surface can be quantified using average wear width, depth, and wear rate, as shown in Figure 12. From both macroscopic and localized 3D perspectives, the substrate exhibits the most severe wear, displaying wider and deeper wear tracks and pits. The average wear width of the substrate is 983.57 μm, with an average wear depth of 13.59 μm. This may be due to the absence of hard alloy phases in the substrate to resist the plastic deformation induced by micro-contact points.

In comparison, the samples prepared at scanning speeds of 4, 6, and 8 mm/s have wear widths of 443.96, 597.42, and 734.20 μm, and average wear depths of 8.63, 10.81, and 13.28 μm, respectively. The wear track width and depth of the coating samples are significantly smaller than those of the substrate. In the initial wear stages, the coating exhibits high strength, making it difficult for the counterpart material to significantly reduce the middle of the wear track, which instead gradually wears against the coating. The smaller width of the wear tracks demonstrates that the coating offers superior wear resistance compared to the substrate.

Wear rate (*W*) is an important metric for evaluating material wear resistance and can be calculated using the following formula:

$$W=\frac{V}{FL}$$

where *V* represents the wear volume (mm^3^), *F* is the applied load (N), and *L* is the total sliding distance (m).

Figure 12b shows the wear rates of the substrate and the coatings prepared at different scanning speeds. The wear rate of the substrate is 1.2 × 10^−4^ mm^3^/N·m, while the wear rates of the coatings with scanning speeds of 4, 6, and 8 mm/s are 3.9 × 10^−5^, 8.5 × 10^−5^ and 9.6 × 10^−5^ mm^3^/N·m, respectively. The wear rate of the coatings is one order of magnitude smaller than that of the substrate. And the wear rate of the coatings prepared at a scanning speed of 4 mm/s is the smallest, which is due to the good metallurgical bond between the coatings and the substrate. The formation of a good metallurgical bond between the coating and the substrate and the coating containing more compound phases (V_8_C_7_, Cr_7_C_3_) with a higher hardness play an important role in decreasing the wear rate.

**Figure 13:**
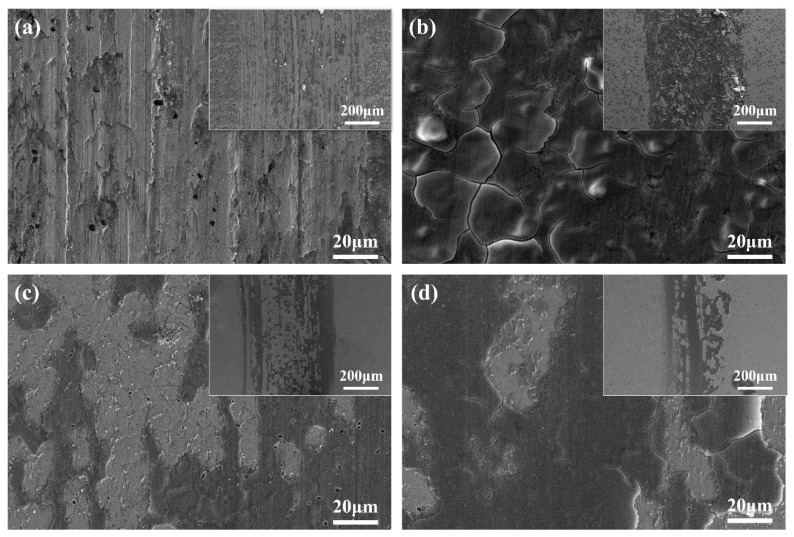
Wear morphology of (**a**) 60Si2Mn and Rockit 606 cladding prepared using different scanning speeds: (**b**) 4, (**c**) 6, and (**d**) 8 mm/s.

To gain a clearer understanding of the wear mechanisms of the cladding coating and the substrate, the wear marks were characterized microscopically. Figure 13a and Figure 13b–d show the SEM images of the surface wear marks of the 60Si2Mn substrate and coatings prepared at different scanning speeds, respectively.

Figure 13a presents the surface wear marks of the 60Si2Mn substrate, showing deep furrows and a significant amount of dark adhesive material. When the counterpart material comes into contact with the wear surface, the surface oxide film or contamination film breaks down, exposing fresh metal surfaces. Moreover, the heat generated by friction cannot dissipate quickly, resulting in local temperature increases and intensified wear. Under the complex alternating loads and cyclical shear stresses, severe plastic deformation occurs on the material’s local surface, leading to dislocation accumulation and stress concentration in the surface and subsurface coatings. This may induce the production and initiation of cracks. As wear continues, the cracks propagate under alternating stress, eventually leading to surface spalling and the formation of pitting. Some wear debris gets compressed and bonded onto the friction sample’s surface due to adhesive or welding-like mechanisms. As the wear process progresses, both the counterpart material and the substrate experience wear, leading to an increase in the contact area and a decrease in stress. However, due to the elevated temperatures at the wear site, fatigue may cause localized delamination of the substrate, leading to pitting. The worn portions of the counterpart material and substrate mix together, moving with the counterpart material. The wear debris increases over time and gradually adheres to the substrate, forming the dark adhesive material. EDS analysis reveals that the dark adhesive material contains higher levels of oxygen and silicon (O14.75, Si20.99), indicating the presence of oxidative wear. These results suggest that during the wear of the substrate, oxidative wear and adhesive wear predominantly occur, accompanied by minor fatigue wear.

In Figure 13b–d, the surface wear marks of coatings prepared at different scanning speeds are shown. When the scanning speed is 4 mm/s, micro cracks and pores are generated at the wear marks, mainly due to the addition of V_8_C_7_ and Cr_7_C_3_, which increase the stress of the coating and form cracks. Due to the presence of more dispersed V_8_C_7_ and Cr_7_C_3_ in the coating, the hardness and strength of the coating are increased, and the fusion layer at the joint is difficult to pull off. Therefore, the fusion layer does not produce plows or pits, and the main wear forms are oxidation wear and a small amount of adhesive wear. As the scanning speed increases, the heat input decreases and the generation of strengthening phases decreases. During the wear process, the coating will experience local cracking and damage, and the broken coating will slide along with the grinding pair, causing wear to the silicon film layer. Therefore, the main forms of wear are oxidation wear and peeling wear, accompanied by a small amount of abrasive wear.

### 3.5. Corrosion Behavior

**Figure 14:**
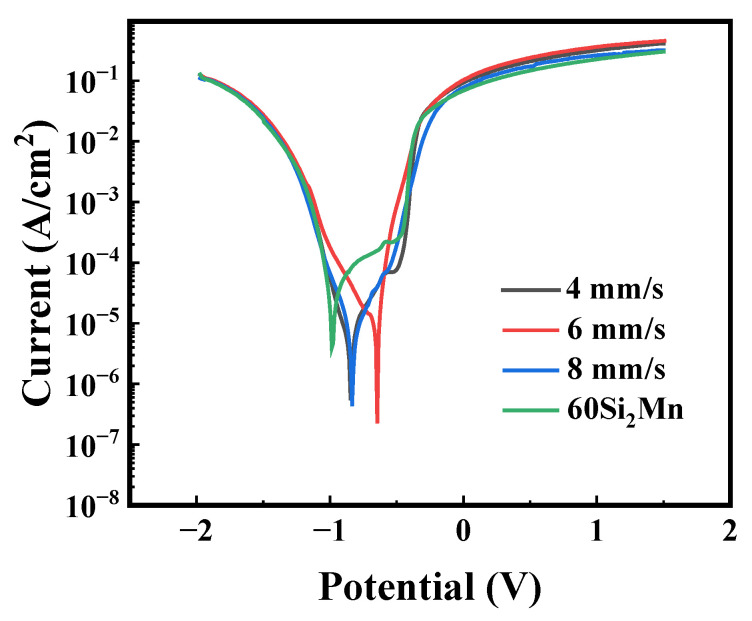
Tafel slopes of the 60Si2Mn and Rockit 606 cladding samples.

Figure 14 and Table 4 show the results of electrochemical tests performed on the substrate and Rockit 606 alloy coating. As shown in Figure 14, the self-corrosion potential (E) of the 60Si2Mn substrate was −0.986 V, and the corrosion current density (i) was 3.956 × 10^−6^ A∙cm^−2^. The observed self-corrosion potentials of the coatings prepared at scanning speeds of 4, 6, and 8 mm/s were −0.845, −0.645, and −0.834 V, respectively. The corresponding corrosion current densities (i) were 5.417, 2.243, and 4.308 × 10^−7^ A∙cm^−2^, respectively. These values were significantly lower than the self-corrosion current density of the substrate (3.956 × 10^−6^ A∙cm^−2^), indicating that the corrosion resistance of the substrate was significantly enhanced by the presence of the coating. The enrichment of Cr and Mo elements in the coating makes it very easy to form a passivation film rich in Cr and Mo oxides, which improves the passivation and local corrosion resistance of the coating and enhances the corrosion resistance of the coating.

## 4. Conclusions

1. The Rockit 606 alloy coatings are prepared by laser cladding under scanning speeds. There is a clear transition zone between 60Si2Mn steel substrate and Rockit 606 alloy coatings. There are no obvious cracks and pores in the coatings. The microstructure Rockit 606 alloy coating consists of γ-Fe, V_8_C_7_, and Cr_7_C_3_.
2. The microhardness of the Rockit 606 alloy coatings decreases with an increase in the scanning speed. When the scanning speed is 4 mm/s, the highest microhardness value reaches 867.2 HV, which is about three times of that of the substrate.
3. The average coefficients of friction (COFs) of the coatings decreased with an increase in the scanning speed, which led to the corresponding decrease of the wear rate. The best wear resistance of the prepared coatings was obtained at a scanning speed of 4 mm/s. When the scanning speed is 4 mm/s, the wear behavior of the coating is mainly oxidative wear and a small amount of adhesive wear. With the increase in scanning speed, the wear forms become oxidative wear and peeling wear, accompanied by a small amount of abrasive wear.
4. The self-corrosion current density of the coatings prepared by laser cladding in 3.5 wt.% NaCl solution is one order of magnitude lower than that of the substrate, indicating that the coatings have better corrosion resistance properties.

## Figures and Tables

**Table 1:** Nominal chemical composition of 60Si2Mn and Rockit 606 (45–180 μm) (wt.%).

Component	C	Si	Mn	P	S	Cr	Ni	Cu	Mo	B	V	Fe
60Si2Mn	0.56~0.64	1.5~2.0	0.7~1.0	≤0.035	≤0.035	≤0.035	≤0.035	≤0.025	-	-	-	Bal.
Rockit 606	1.9	1.1	0.5	-	-	4.7	-	-	0.6	0.8	6.0	Bal.

**Table 2:** Experimental parameter table.

Serial Number	Substrate Material	Powder Used	Laser Beam Power (W)	Scanning Speed (mm/s)
1	60Si2Mn	Rockit 606	1600	4
2				6
3				8

**Table 3:** Chemical compositions of points D~F (%, atom fraction).

Point	B	C	Si	V	Cr	Mn	Fe	Mo	Possible Phase
D	0.85	10.31	2.76	1.76	1.98	0.30	81.96	0.09	γ-Fe
E	0.15	13.72	1.49	3.26	4.30	0.99	75.15	0.36	Cr_7_C_3_, V_8_C_7_
F	0.08	37.82	0.52	43.55	1.30	0.30	16.13	0.29	V_8_C_7_

**Table 4:** Polarization parameters of 60Si2Mn and Rockit 606 cladding samples.

Sample	Self-Corrosion Potential (V)	Corrosion Current Density (A/cm^2^)
60Si2Mn	−0.986	3.956 × 10^−6^
4 mm/s	−0.845	5.417 × 10^−7^
6 mm/s	−0.645	2.243 × 10^−7^
8 mm/s	−0.834	4.308 × 10^−7^

## Data Availability

The original contributions presented in the study are included in the article; further inquiries can be directed to the corresponding authors.
